# Myth-Busting the Zone-of-Injury Concept: A Prospective Study on the Vascular Response to High-Energy Lower Extremity Trauma

**DOI:** 10.1097/PRS.0000000000010980

**Published:** 2023-08-10

**Authors:** Adas Cepas, Juha Kiiski, Marja Majava, Ivana Kholová, Ilkka Kaartinen

**Affiliations:** Tampere, Finland; and Kaunas, Lithuania; From the 1 Department of Musculoskeletal Surgery and Diseases, Tampere University Hospital; 2 University of Tampere, Faculty of Medicine and Health Technology; 3 Department of Plastic and Reconstructive Surgery, Hospital of Lithuanian University of Health Sciences, Kaunas Clinics; 4 Department of Pathology, Fimlab Laboratories.

## Abstract

**Background:**

Although the zone-of-injury concept is widely accepted, no histologic studies of vessel wall changes causing the phenomenon have been reported. In this prospective study, the vascular response to high-energy lower extremity trauma was investigated to evaluate the validity of the zone-of-injury concept.

**Methods:**

The histologic appearance of arterial and venous walls in the zone of injury was studied in 19 patients (median age, 46 years; interquartile range, 29.5 to 62.5 years) who underwent osteosynthesis and free flap reconstruction after high-energy lower extremity open fracture. Vascular samples were harvested from the injured extremity, and control samples were harvested from the free flap donor site. Histologic and morphometric characteristics of the vessels were analyzed microscopically and using digital pathology QuPath software.

**Results:**

Vascular samples were harvested on postinjury days 1 through 11. Intimal thickness was more than 3 times greater in arteries harvested from the zone of injury than in control samples (*P* < 0.01), and the intima/media ratio was 2-fold that in control samples (*P* = 0.01). Arterial intimal fibrosis was more evident in vessels harvested from the zone of injury (*P* < 0.01), but medial fibrosis and medial thickness did not differ significantly between groups. Venous intimal thickening (*P* < 0.01) and the intima/media ratio (*P* = 0.02) were greater in samples from the zone of injury. Fibrosis-related changes did not differ between groups (*P* = 0.45).

**Conclusions:**

These findings support the validity of the zone-of-injury concept by providing a novel histologic basis for this phenomenon. Intimal thickening and arterial intimal fibrosis are prominent histologic features of vessels affected by major lower extremity trauma.

In reconstructive plastic surgery of the lower extremities, the zone of injury refers to the area surrounding the injured tissue, where reactive changes occur because of the inflammatory response.^1^ This concept was established in the early 1980s to explain the 20% to 30% flap failure rate encountered in lower extremity free flap surgery.^2^ Pioneers in this field considered that blood vessels in the zone of injury are more prone to vasospasm and lack the thromboresistant properties of healthy vessels,^2,3^ and thus recommended that surgeons avoid performing the anastomosis in this area because of an increased risk of flap failure. This notion has guided plastic surgeons to perform the anastomosis proximal to the injury site, or outside the zone of injury. Although this is a well-known principle among plastic surgeons, its biologic basis has not been unequivocally described.^4^ Moreover, the assumed safe distance from the injury site to the anastomoses has decreased from earlier long incisions and vein grafts to just a few centimeters above the bony fracture site.^1,5–10^

Despite wide acceptance of the zone-of-injury concept, histologic studies of the vessel wall changes causing the phenomena have not been reported. In addition to the absence of studies in humans, few animal model studies on arterial wall remodeling after endovascular injury have been published.^11–14^ These studies described 3 different pathways of intimal thickening secondary to increased myofibroblast and medial smooth muscle cell activity in the intimal layer. The mechanism and extent of vascular injury in these animal model studies, however, are not comparable with those of high-energy blunt trauma of the lower extremity.

We investigated the relevant histologic changes of arterial and venous walls in the zone of injury after high-grade open fracture of the lower extremity.

## PATIENTS AND METHODS

This prospective study investigating human vessel walls was conducted at Tampere University Hospital and Tampere University, Finland. A total of 19 patients who sustained high-energy open fractures of the lower extremities requiring free flap reconstruction were included: 16 open tibial fractures (IIIB or worse), 1 open femur fracture (IIIB), and 2 open fractures of the foot. The patients were operated on at Tampere University Hospital between March of 2020 and October of 2022 according to the institutional algorithm on open lower extremity fracture management. Except for 5 multitrauma cases with concomitant injuries that were not fit for reconstruction during the first week, patients with acute open fractures underwent initial débridement and bony stabilization within 24 hours of hospital admission, followed by definitive fixation and soft-tissue reconstruction within a week.

**Fig. 1.**
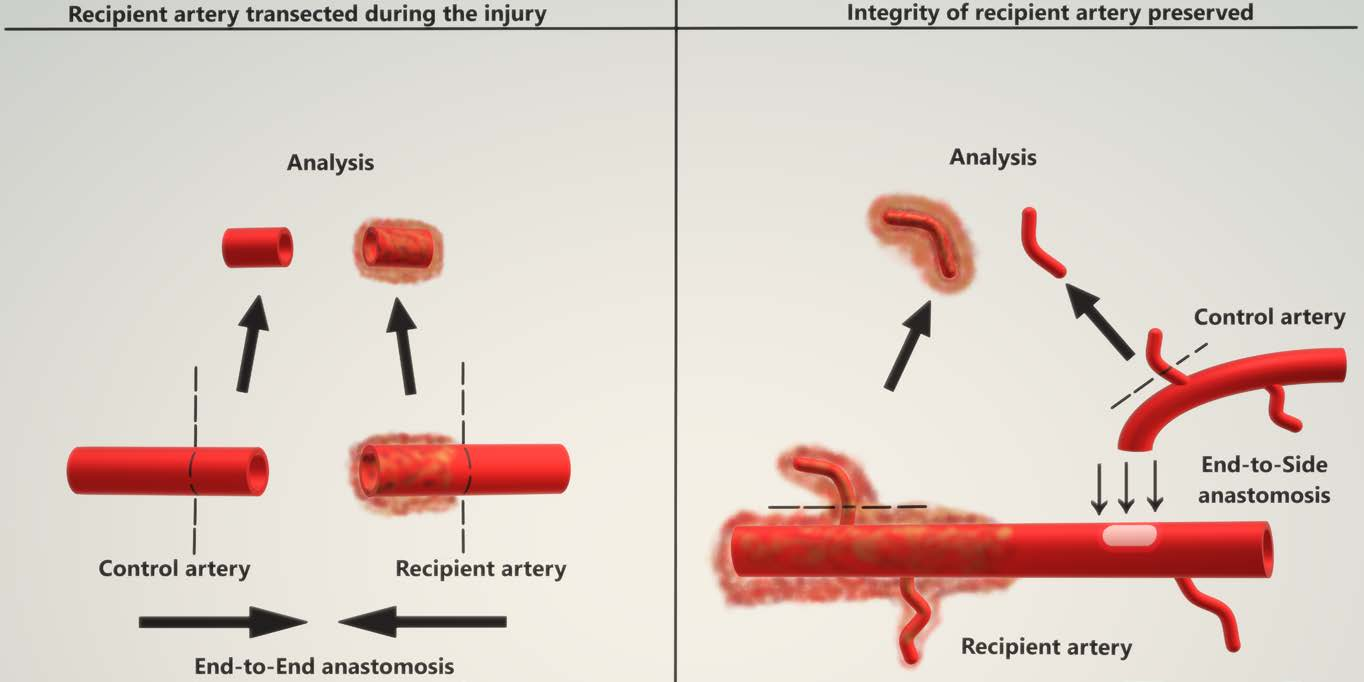
Harvest of arterial samples before anastomosis of the free flap. (*Left*) Sample harvest procedure and anastomosis in arteries that were transected during the injury. (*Right*) Anastomosis and sample harvest procedure if the arterial integrity was preserved.

At the time of the soft-tissue reconstruction, 1 arterial and 1 venous biopsy sample from the injury site of traumatized lower extremity and 1 venous and arterial control sample from the free flap donor site were collected from each patient. In case the recipient artery was transected during the injury, the arterial stump at the level of injury was trimmed and harvested for the study, and end-to-end anastomosis was performed several centimeters proximal to the injury site. If the integrity of the recipient artery was preserved, a side branch of recipient vessel was harvested from the injury site, and end-to-side anastomosis was performed proximally, based on clinical judgment of recipient vessel quality and patency of the flow (Fig. 1). Venous anastomoses were always performed end-to-end, allowing for sample harvest at the most affected zones. Control samples harvested from the free flap donor sites were flap pedicle vessels or their side branches that were size-matched to the vessels harvested from the zone of injury.

### Histologic Analysis

**Fig. 2.**
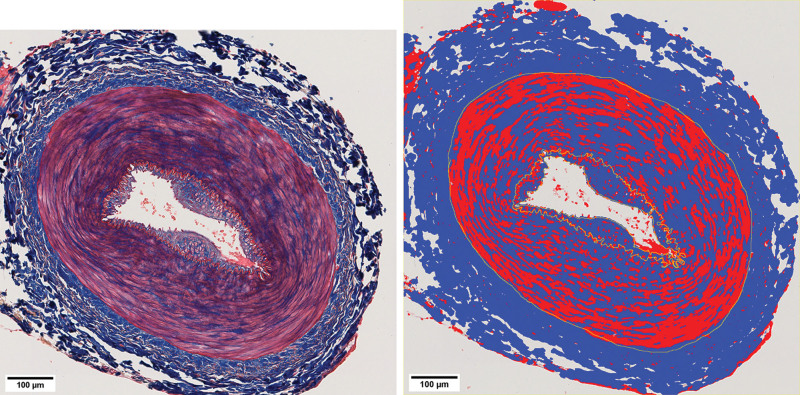
Measuring vessel wall fibrosis in Masson trichrome–stained sections. (*Left*) Arterial section with annotated intimal and medial layers. (*Right*) The same image after application of the pixel classifier function (*blue* represents collagen fibers).

Vessel wall samples were fixed in 10% formalin at room temperature for 24 hours and further processed to paraffin blocks. Slides of 5-µm-thick vessel walls were prepared and stained with hematoxylin & eosin, Elastic Stain Kit (Verhoeff Van Gieson; Abcam, Cambridge, MA), and Masson trichrome (Sigma; Merck Life Science, Espoo, Finland) for histologic analysis. The histologic slides were scanned (NanoZoomer S360; Hamamatsu Photonics, Hamamatsu City, Japan). Both microscopy and the open-source digital pathology software QuPath were used for pathology image analysis.^15^ Intimal and medial thicknesses were measured on elastic Van Gieson–stained slides at the thickest part of the vessel wall circumference using the “line” feature. Interstitial fibrosis of intimal and medial layers was quantified by the semiautomated artificial intelligence–guided pixel classifier function. The classifier was trained on multiple images before its application for the analysis. To detect fibrosis and smooth muscle cells, specific thresholds were set for blue (collagen) and red (smooth muscle cells) stained components in Masson trichrome–stained sections, as shown in Figure 2. The fibrosis of the vessel walls was calculated as a percentage of the annotated intimal or medial area.

### Statistical Analysis

Quantified data are presented as median and interquartile range (IQR). Proportions are given as percentage or ratio. The Mann-Whitney *U* test was used to test statistical significance for continuous variables. A *P* value of 0.05 was considered statistically significant. All statistical analyses were performed using SPSS statistics 24.0 (IBM, Armonk, NY).

## RESULTS

### General Characteristics of the Study Population

The median age of the patients included in the study was 46 years (IQR, 29.5 to 62.5 years), with 84% being men and 16% women. Of the 19 patients, 21% had cardiovascular comorbidities and 26% were smokers. The median time frame from injury to definitive fixation and soft-tissue reconstruction was 6 days (IQR, 3.5 to 8.5 days), with 79% of the patients operated on within the first 7 days after trauma. The remaining 5 patients had multiple traumas with concomitant injuries, and underwent reconstruction on postinjury days 8 through 11. The most common external cause of injury was road traffic accident (42%), followed by a fall from height (37%) and forestry or heavy industry–related trauma (21%). (**See Table, Supplemental Digital Content 1**, which provides case-by-case data regarding demographics, flap reconstructions, and vessels harvested for the analysis, http://links.lww.com/PRS/G820.)

### Morphometric Characteristics of the Arterial Walls

**Fig. 3.**
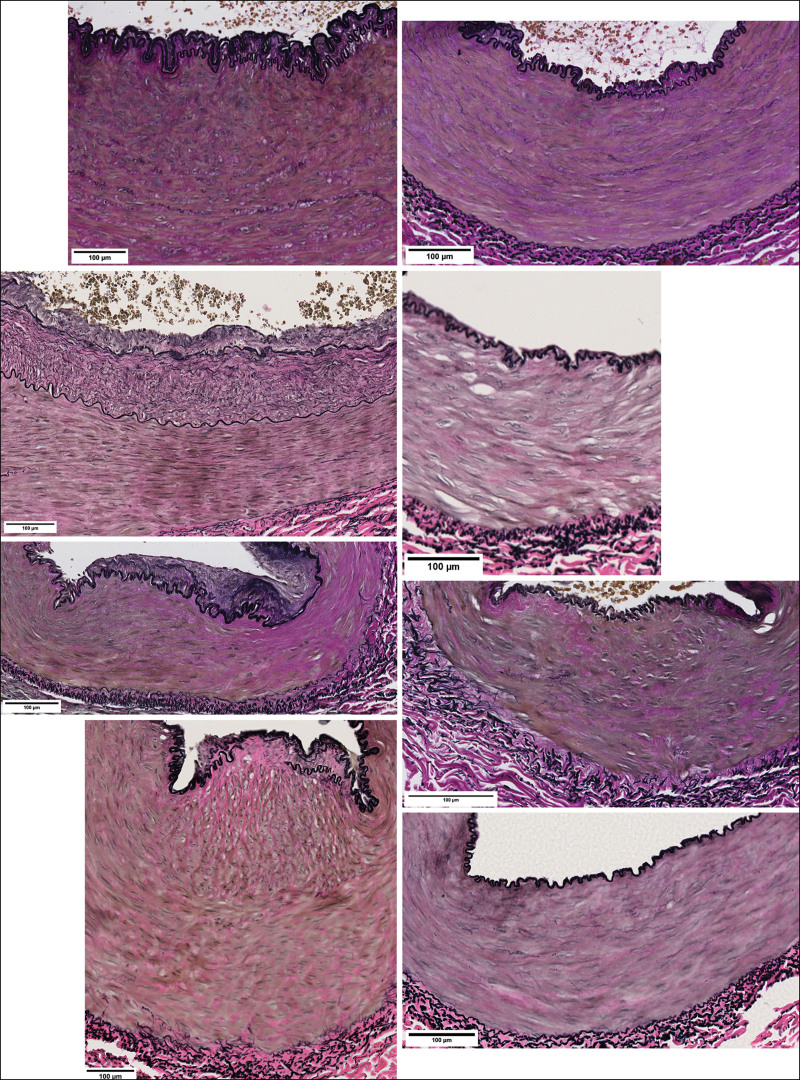
Postinjury dynamics of arterial intimal thickening. Photographs represent paired arterial-wall sections harvested from the zone of injury (*left*) and controls (*right*) at different time points after injury. Samples harvested on postinjury days 1 (*above*), 3 (*second row from above*), 6 (*second row from below*), and 10 (*below*).

**Fig. 4.**
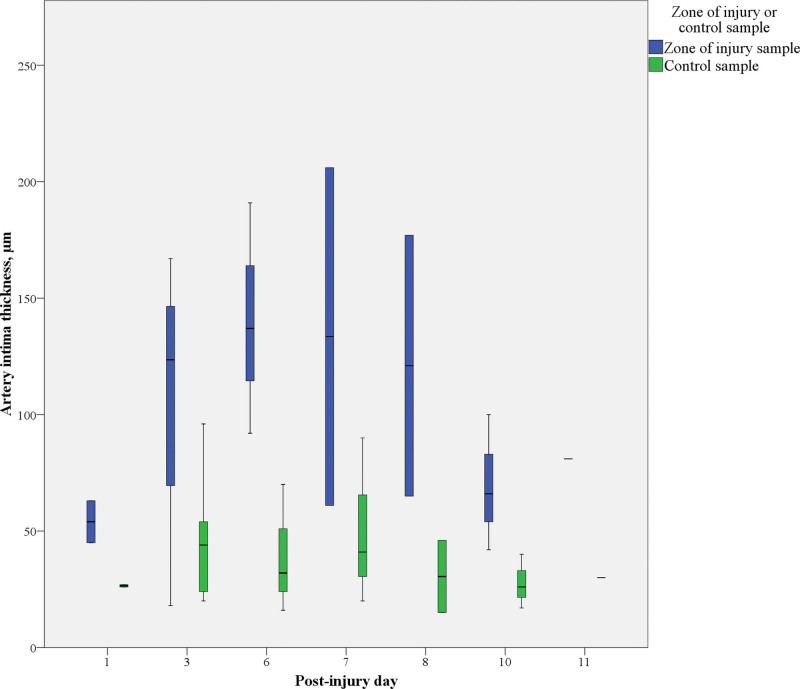
Dynamics of arterial intimal thickening.

**Fig. 5.**
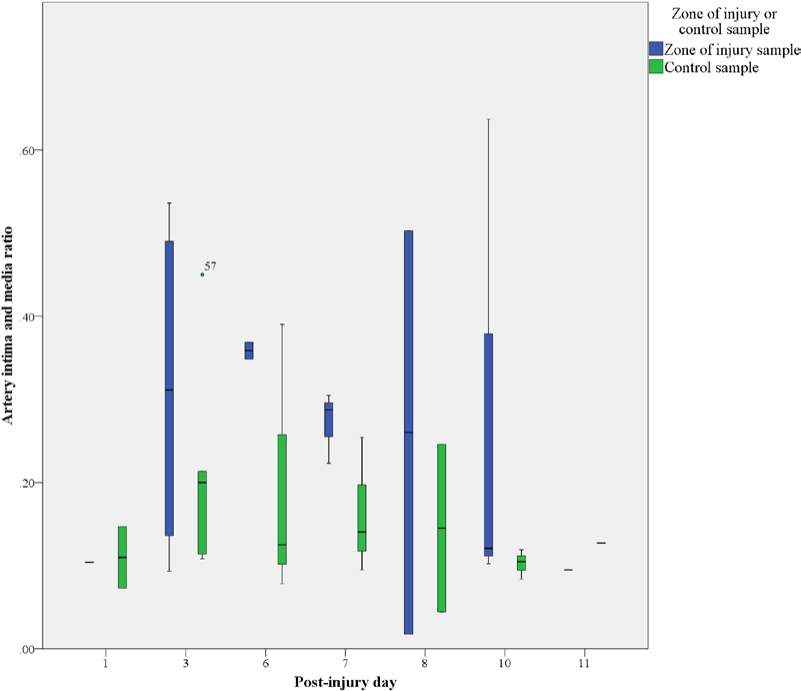
Arterial intima/media ratio and its relation to the day of sampling.

Histologic examination of the arterial walls revealed broad intimal hyperplasia in the zone of injury and control samples (18 to 206 µm versus 15 to 96 µm, respectively). The median intimal thickness of arteries harvested from the zone of injury, however, was more than 3 times greater than that in control samples (Table 1). Moreover, in the zone of injury, we observed a trend toward arterial intimal thickening related to the postinjury day of sampling (Fig. 3). Arterial intimal hyperplasia was mild in samples collected from the zone of injury on the first postinjury day, but already present in samples collected on postinjury day 3. The most prominent arterial intimal thickening was observed in samples harvested on days 6 and 7 after trauma, with a gradual decrease in intimal thickness in samples collected later. In contrast to these findings, median intimal thickness in the control samples remained unchanged regardless of the date of sampling or interindividual differences (Fig. 4). Although the arterial media thickness did not significantly differ between the zone of injury and control samples (*P* = 0.11), the intima/media ratio was higher in the zone of injury samples (*P* = 0.01) (Fig. 5). Despite the wide variation in arterial intimal fibrosis in the zone of injury (24% to 83%) and control (5% to 92%) samples, intimal fibrosis was more evident in arteries harvested from the zone of injury, with median fibrosis of 70% (IQR, 60% to 80%) compared with 39% (IQR, 21% to 55%) in control samples (*P* = 0.01). Although median fibrosis of the arterial media was 51% (IQR, 39% to 63%) in the zone of injury and 42% (IQR 31% to 54%) in control samples, the difference was not statistically significant (*P* = 0.1).

**Table 1.** Morphometric Characteristics of Arterial Walls in the Zone of Injury and Control Samples^a^

Variable	Zone of Injury (µm)	Control (µm)	*P*
Artery intimal thickness	92 (47–137)	30 (17–43)	<0.01
Artery medial thickness	320 (162–478)	235 (169–301)	0.11
Arterial intima/media ratio	0.26 (0.09–0.43)	0.12 (0.08–0.17)	0.01

a Data are presented as mean (interquartile range).

There were no significant differences between the arterial walls of different donor sites. Recipient arteries in cases reconstructed with latissimus dorsi (LD) flaps or gracilis/anterolateral thigh (ALT) flaps showed no significant differences. (**See Table, Supplemental Digital Content 2**, which shows morphometric characteristics of control sample arterial walls and recipient arteries in LD and gracilis/ALT flap reconstructions, http://links.lww.com/PRS/G821.)

### Morphometric Characteristics of the Venous Walls

**Fig. 6.**
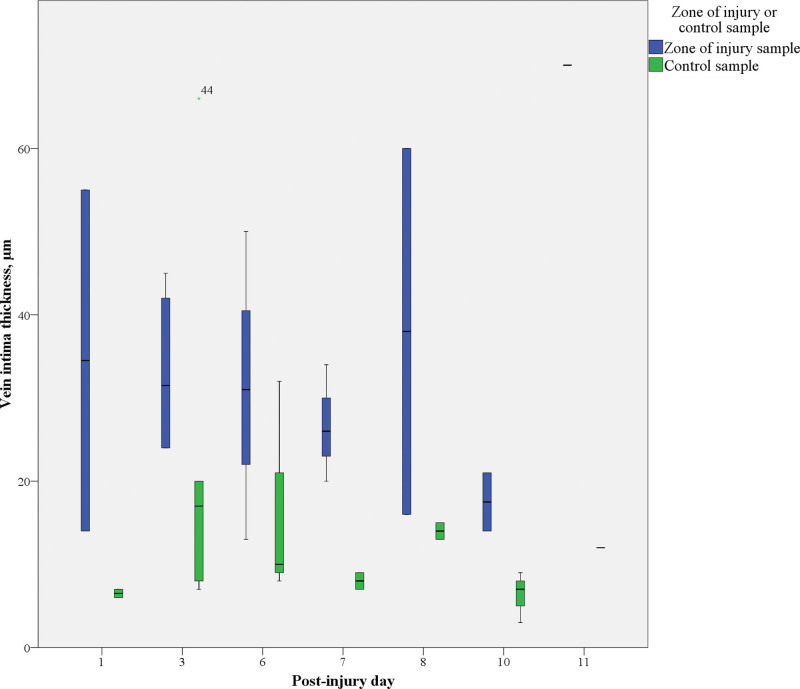
Venous intimal thickening and its relation to the postinjury day of sampling.

In contrast to the arteries, venous intimal hyperplasia was more evenly distributed between the zone of injury and control samples (13 to 70 µm versus 3 to 66 µm). Despite the similar distribution range, the median venous intimal thickness was more than 2 times greater in the zone of injury than in the controls (*P* < 0.01) (Table 2). In veins, unlike arteries, the median intimal thickness remained similar throughout the first 6 postinjury days, with some outliers causing peaks on postinjury days 8 and 11 and valleys on postinjury days 7 and 10 (Fig. 6). As with arteries, venous medial thickness did not differ significantly between the zone of injury and control samples (*P* = 0.23), but the intima/media ratio was higher in samples harvested from the zone of injury (*P* = 0.01). Combined intimal and medial venous fibrosis ranged between 25% and 74% in the zone of injury and 23% and 85% in control samples, but no significant differences in the median intimal and medial fibrosis were detected between groups (*P* = 0.45).

**Table 2.** Morphometric Characteristics of Venous Walls in the Zone of Injury and Control Samples^a^

Variable	Zone of Injury (µm)	Control (µm)	*P*
Vascular histology			
Vein intimal thickness	24 (6.5–41.5)	9 (5–13)	<0.01
Vein medial thickness	152 (69–235)	139 (84–194)	0.23
Vein intima/media ratio	0.21 (0.14–0.28)	0.07 (0.01–0.13)	0.02
Fibrosis			
Vein intimal and medial fibrosis (combined)	39 (26–52)	48 (41–53)	0.45

a Data are presented as mean (interquartile range) (histology) or % (interquartile range) (fibrosis).

The medial thickness of the LD flap pedicle vein was greater than that of the gracilis/ALT pedicle vein (*P* = 0.03). Otherwise, no significant differences in vein wall morphometrics were observed. (**See Table, Supplemental Digital Content 3**, which shows morphometric characteristics of control sample venous walls and recipient veins in LD and gracilis/ALT flap reconstructions, http://links.lww.com/PRS/G822.)

### Clinical Outcomes

All vascular anastomoses were performed under microscope magnification. No free flap losses or takebacks because of anastomotic problems were encountered in the study series. One patient required a kickstand frame to off-load and manage position-induced venous congestion, after which the flap healed uneventfully. No cases of deep infection or nonunion occurred, but the follow-up period was short, ranging from 3 to 33 months. No major amputations were required.

## DISCUSSION

Existing literature on the zone-of-injury concept is based on observational studies and subjective interpretations addressing the quality of free flap recipient vessels in traumatized lower extremities. Although the concept was first described in the 1980s, it lacks biologic validity. This study was the first clinical study to evaluate histologic changes of the blood vessels affected by high-energy lower extremity trauma and provides the biologic basis of the zone-of-injury concept.

The most prominent finding in this study was vascular intimal hyperplasia, which was obvious in the samples harvested from the zone of injury. Our study data revealed that the vascular response to trauma had a sudden onset and resulted in intimal hyperplasia, which was particularly evident in arteries harvested on days 3 to 7 after injury. These changes, however, were not as pronounced in veins. (**See Figure, Supplemental Digital Content 4**, which shows evident arterial intimal thickening in the zone of injury sample [*left*] harvested on day 10 after injury compared with a control sample [*right*] from a 31-year-old patient without concomitant comorbidities, http://links.lww.com/PRS/G823.) In support of our study findings, intimal hyperplasia was reported in previous experimental animal model studies after simulated endovascular arterial injury.^11–14^ Mechanical injury induced by the conventional balloon angioplasty procedure led to a shift of vascular smooth muscle cells (VSMCs) from a contractile to a synthetic phenotype in the arterial media.^12^ These synthetic VSMCs are present in the intima several days after vascular trauma and play a key role in intimal thickening by active migration, proliferation, and extracellular matrix secretion.^12,14^ The arterial response to thermal injury seems to be different, although it also results in intimal thickening. In an experimental animal model study, monocytes and macrophages populated the neointima at early time points after thermal endovascular injury, but at 4 weeks after injury, the thickened neointima was mostly populated with myofibroblasts and collagen fibers.^11^ Bayes-Genis et al.^11^ hypothesized that blood-derived monocytes and macrophages are progenitors of collagen-secreting neointimal myofibroblasts rather than medial VSMCs or adventitial myofibroblasts, as reported in previous studies.^11–13^ Although intimal thickening mechanisms reported in animal model studies might also be valid in human vascular remodeling, our study results showed a more rapid vascular response to injury than described in the aforementioned animal model studies.

Another important finding regarding vessel wall quality was vessel wall fibrosis. Compared with control vessels, arteries harvested from the zone of injury were more fibrotic, although the time from the injury to vessel harvest ranged from 1 to 11 days. High extracellular matrix deposits in the arterial intima suggest the presence and increased activity of profibrotic cells such as myofibroblasts or fibroblasts. Although the incidence of arterial intimal fibrosis in samples harvested from the zone of injury was almost 2-fold greater than in the control samples (*P* = 0.01), those changes were not evident in the arterial media (*P* = 0.1). In veins, combined medial and intimal fibrosis exhibited a lower trend in samples harvested from the zone of injury compared with controls (39% versus 48%; *P* = 0.45), leading to a question of whether the onset of intimal fibrosis in the veins is slower, different, or nonexistent.

In our study, the control samples were harvested from free flap pedicles of 2 different anatomic regions: trunk (LD flap) and thigh (gracilis or ALT flap). One might suspect that vessel wall thickness is a consequence of the anatomic site. However, there were no significant differences noted between trunk and thigh flap arterial walls. This suggests that the vessel wall changes we described in this article are relevant to the zone-of-injury concept and are in fact caused by the high-energy trauma.

Although our study findings show gradual decrease in arterial intimal thickening past day 7 after the injury, our study did not investigate endothelial function.

There is no clinical evidence suggesting that postponing reconstruction would result in better clinical or flap-related outcomes.^16^ On the other hand, delay in reconstruction is known to increase the risk of bony nonunion, deep infection, and major amputation rates, and current guidelines advocate that the final soft-tissue reconstruction should be performed ideally within 72 hours after the injury.^17^

This study has several strengths. First, it is prospective in nature and is the first to describe the zone-of-injury concept from the vascular histology aspect. Second, we were able to compare the vascular morphology of injured vessels and healthy control vessels from the same patient, thereby avoiding interindividual differences. Third, variations in vessel harvest time points allowed us to define the dynamics of the histologic changes in the vessel walls that seemed to be logical according to normal tissue remodeling after injury and findings from previous animal studies.

This study also has limitations. First, because of the rarity of this type of trauma, the sample size was relatively small. Second, the heterogeneity of the patients regarding age, sex, comorbidities, and other interindividual differences might affect the vascular wall histologic findings. Third, because the extent of the zone of injury and the severity of the soft-tissue injuries varied among patients, it is possible that some samples were harvested from more severely injured sites compared with others, and this could affect the extent of the vascular response, as observed by Shi et al.^13^

No flap losses were encountered after soft-tissue reconstructions of severely injured lower extremities. Further studies at the cellular and molecular levels are needed to define the exact mechanisms of vascular intimal hyperplasia and especially how it might affect vascular patency in free flap surgery in the setting of lower extremity trauma.

## CONCLUSIONS

Our study findings support the validity of the zone-of-injury concept. The most prominent histologic features of the vessels affected by major lower extremity trauma are intimal thickening and arterial intimal fibrosis. Despite these histologic findings in affected vessels, excellent free flap outcomes were achieved by placing the anastomoses based on clinical judgment of the vessel quality.

## DISCLOSURE

The authors have no financial interests to disclose.

## ACKNOWLEDGMENTS

Dr. Kaartinen received a grant (no. 9AB018) from Competitive State Research Financing of the Expert Responsibility Area of Tampere University Hospital, which was used to cover expenses related to the preparation and processing of histologic slides as well as language-editing service.

## Supplementary Material


