# Supplementary material for: Myth-Busting the Zone-of-Injury Concept: A Prospective Study on the Vascular Response to High-Energy Lower Extremity Trauma
Source: Plast Reconstr Surg. 2023 Aug 10;154(1):190–8. doi: 10.1097/PRS.0000000000010980 (PMC11195921; doi:10.1097/PRS.0000000000010980)
Supplement: Supplementary file 1 [file prs-154-190e-s001.pdf]

Table, Supplemental Digital Content 1. Detailed characteristics of the study population.

Case	Age	Comorbidities, smoking status	Reconstruction and vessel harvest day (post-injury)	Injury	Free flap	Zone of injury sample origin	Control sample origin
**1**	62	Smoker	6	Open tibial fracture IIIB	Latissimus dorsi	Posterior tibial	Thoracodorsal pedicle
**2**	56	-	7	Open tibial fracture IIIB	Latissimus dorsi	Anterior tibial	Thoracodorsal pedicle
**3**	29	-	11	Open tibial fracture IIIB	Gracilis	Posterior tibial	Gracilis pedicle
**4**	48	Smoker	3	Open tibial fracture IIIB	Gracilis	Posterior tibial	Gracilis pedicle
**5**	62	-	6	Open tibial fracture IIIB	Gracilis	Posterior tibial	Gracilis pedicle
**6**	44	Asthma	3	Open tibial fracture IIIB	Latissimus dorsi	Posterior tibial	Thoracodorsal pedicle
**7**	44	Arterial hypertension, diabetes type I	6	Open tibial fracture IIIB	Gracilis	Posterior tibial	Gracilis pedicle
**8**	33	-	1	Open calcaneal fracture	ALT	Posterior tibial	Descending branch of lateral circumflex femoral artery
**9**	66	-	8	Open tibial fracture IIIB	Latissimus dorsi	Posterior tibial	Thoracodorsal pedicle
**10**	75	Coronary artery disease	3	Open tibial fracture IIIB	Gracilis	Posterior tibial	Gracilis pedicle
**11**	78	Persistent atrial fibrillation	7	Open tibial fracture IIIB	Latissimus dorsi	Posterior tibial	Thoracodorsal pedicle
**12**	29	Smoker	10	Open tibial fracture IIIC	Latissimus dorsi	Medial superior genicular	Thoracodorsal pedicle
**13**	23	-	10	Open Lisfranc fracture	Latissimus dorsi	Dorsalis pedis	Thoracodorsal pedicle
**14**	48	-	8	Open tibial fracture IIIB	Gracilis	Posterior tibial	Gracilis pedicle
**15**	31	Smoker	10	Open tibial fracture IIIB	Gracilis	Posterior tibial	Gracilis pedicle
**16**	28	-	3	Open tibial fracture IIIB	Latissimus dorsi	Medial sural	Thoracodorsal pedicle
**17**	19	Smoker	1	Open tibial fracture IIIB	Latissimus dorsi	Posterior tibial	Thoracodorsal pedicle
**18**	56	Arterial hypertension	3	Open tibial fracture IIIB	Gracilis	Posterior tibial	Gracilis pedicle
**19**	49	-	7	Open femur fracture IIIB	Latissimus dorsi	Branch of profunda femoris	Thoracodorsal
