# Supplementary material for: Myth-Busting the Zone-of-Injury Concept: A Prospective Study on the Vascular Response to High-Energy Lower Extremity Trauma
Source: Plast Reconstr Surg. 2023 Aug 10;154(1):190–8. doi: 10.1097/PRS.0000000000010980 (PMC11195921; doi:10.1097/PRS.0000000000010980)
Supplement: Supplementary file 2 [file prs-154-190e-s002.pdf]

Table, Supplemental Digital Content 2. Morphometric characteristics of control sample arterial walls and recipient arteries in LD and gracilis & ALT flap reconstructions.

Control sample arteries			
Variable	LD flap pedicle vessels n=10	Gracilis & ALT flap pedicle vessels n=9	p
Artery intimal thickness M (IQR)	36µm (14-50 µm)	27µm (12-42µm)	0.32
Artery medial thickness M (IQR)	240µm (199-281µm)	231µm (124-338µm)	0.36
Arterial intimal fibrosis % (IQR)	35% (13-57%)	42% (20-64%)	0.71
Arterial medial fibrosis % (IQR)	40% (17-63%)	42% (34-50%)	0.69
Recipient arteries			
Variable	LD flap pedicle vessels n=10	Gracilis & ALT flap pedicle vessels n=9	p
Artery intimal thickness M (IQR)	81µm (11-151µm)	96 µm (62-130µm)	0.82
Artery medial thickness M (IQR)	320µm (198-442µm)	332µm (147-517µm)	0.76
Artery intimal fibrosis % (IQR)	62% (38-86%)	70% (61-79%)	0.28
Artery medial fibrosis % (IQR)	42% (30-54%)	51% (36-66%)	0.39
