# Supplementary material for: Myth-Busting the Zone-of-Injury Concept: A Prospective Study on the Vascular Response to High-Energy Lower Extremity Trauma
Source: Plast Reconstr Surg. 2023 Aug 10;154(1):190–8. doi: 10.1097/PRS.0000000000010980 (PMC11195921; doi:10.1097/PRS.0000000000010980)
Supplement: Supplementary file 3 [file prs-154-190e-s003.pdf]

Table, Supplemental Digital Content 3. Morphometric characteristics of control sample venous walls and recipient veins in LD and gracilis & ALT flap reconstructions.

Control sample veins			
Variable	LD flap pedicle vessels n=10	Gracilis & ALT flap pedicle vessels n=9	p
Venous intimal thickness M (IQR)	9 µm (6-12µm)	15 µm (3-27µm)	0.73
Venous medial thickness M (IQR)	172 µm (133-211µm)	97 µm (39-155µm)	0.03
Venous intimal and medial fibrosis % (IQR)	49% (31-54%)	45% (38-53%)	0.61
Recipient veins			
Variable	LD flap pedicle vessels n=10	Gracilis & ALT flap pedicle vessels n=9	p
Venous intimal thickness M (IQR)	29µm (12-46µm)	24µm (8-40µm)	0.61
Venous medial thickness M (IQR)	147µm (69-221µm)	152µm (72-232µm)	0.37
Venous intimal and medial fibrosis % (IQR)	39% (26-52%)	41% (28-54%)	0.73
