# Supplementary figures and images for: Myth-Busting the Zone-of-Injury Concept: A Prospective Study on the Vascular Response to High-Energy Lower Extremity Trauma
Source: Plast Reconstr Surg. 2023 Aug 10;154(1):190–8. doi: 10.1097/PRS.0000000000010980 (PMC11195921; doi:10.1097/PRS.0000000000010980)

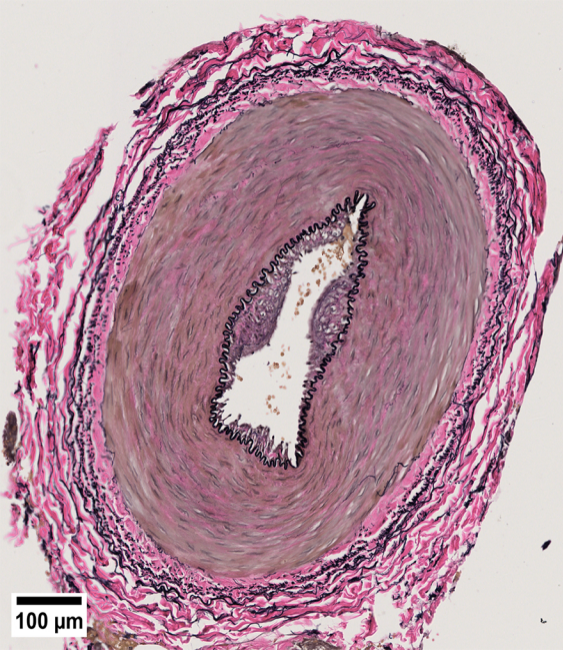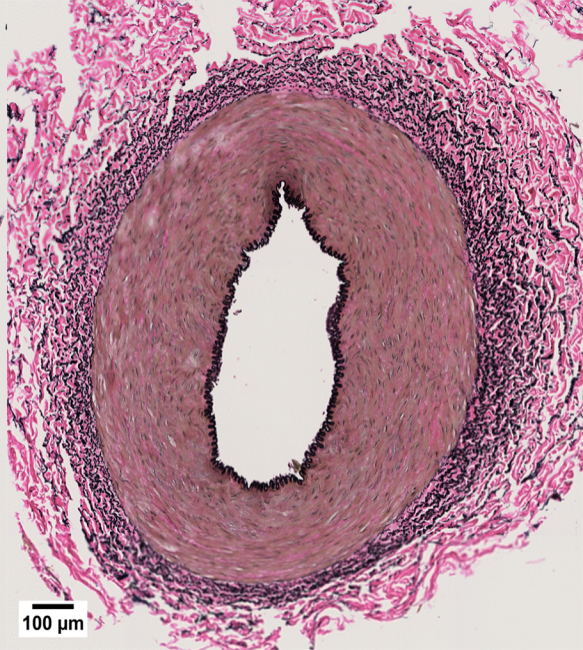

Supplement: Supplementary file 4 [file prs-154-190e-s004.pdf]
